# Experimental HER2-Targeted Therapy Using ADAPT6-ABD-mcDM1 in Mice Bearing SKOV3 Ovarian Cancer Xenografts: Efficacy and Selection of Companion Imaging Counterpart

**DOI:** 10.3390/pharmaceutics14081612

**Published:** 2022-08-02

**Authors:** Javad Garousi, Tianqi Xu, Yongsheng Liu, Olga Vorontsova, Sophia Hober, Anna Orlova, Vladimir Tolmachev, Torbjörn Gräslund, Anzhelika Vorobyeva

**Affiliations:** 1 Department of Protein Science, KTH Royal Institute of Technology, 106 91 Stockholm, Sweden; garousi@kth.se (J.G.); sophia@kth.se (S.H.); 2 Department of Immunology, Genetics and Pathology, Uppsala University, 751 85 Uppsala, Sweden; tianqi.xu@igp.uu.se (T.X.); yongsheng.liu@igp.uu.se (Y.L.); olga.vorontsova@igp.uu.se (O.V.); anzhelika.vorobyeva@igp.uu.se (A.V.); 3 Department of Medicinal Chemistry, Uppsala University, 751 23 Uppsala, Sweden; anna.orlova@ilk.uu.se

**Keywords:** ADAPT, human epidermal growth factor receptor 2, HER2, cancer therapy, DM1, albumin binding domain, engineered scaffold protein

## Abstract

Overexpression of the human epidermal growth factor receptor 2 (HER2) in breast and gastric cancer is exploited for targeted therapy using monoclonal antibodies and antibody-drug conjugates. Small engineered scaffold proteins, such as the albumin binding domain (ABD) derived affinity proteins (ADAPTs), are a promising new format of targeting probes for development of drug conjugates with well-defined structure and tunable pharmacokinetics. Radiolabeled ADAPT6 has shown excellent tumor-targeting properties in clinical trials. Recently, we developed a drug conjugate based on the HER2-targeting ADAPT6 fused to an albumin binding domain (ABD) for increased bioavailability and conjugated to DM1 for cytotoxic action, designated as ADAPT6-ABD-mcDM1. In this study, we investigated the therapeutic efficacy of this conjugate in mice bearing HER2-expressing SKOV3 ovarian cancer xenografts. A secondary aim was to evaluate several formats of imaging probes for visualization of HER2 expression in tumors. Administration of ADAPT6-ABD-mcDM1 provided a significant delay of tumor growth and increased the median survival of the mice, in comparison with both a non-targeting homologous construct (ADAPT_Neg_-ABD-mcDM1) and the vehicle-treated groups, without inducing toxicity to liver or kidneys. Moreover, the evaluation of imaging probes showed that small scaffold proteins, such as ^99m^Tc(CO)_3_-ADAPT6 or the affibody molecule ^99m^Tc-Z_HER2:41071_, are well suited as diagnostic companions for potential stratification of patients for ADAPT6-ABD-mcDM1–based therapy.

## 1. Introduction

Targeted therapy using monoclonal antibodies (mAbs) is well established and extends the survival of patients with disseminated cancer. For example, the monoclonal antibody (mAb) trastuzumab, which targets human epidermal growth factor receptor 2 (HER2), has proven efficacy against HER2-positive breast and gastric cancer and is used routinely in clinics [1]. Furthermore, the anti-epidermal growth factor receptor (EGFR) mAb cetuximab, used for treatment of metastatic colorectal cancer, improves the overall survival of patients with wild-type K-ras tumors [2,3]. Despite these successful examples, the efficacy of mAb-based therapy is limited by the mode of action. Antibodies provide both cytostatic and cytotoxic action; however, antibody-dependent cell cytotoxicity (ADCC) is mediated by immune cells, which could be compromised if a patient undergoes chemotherapy [4]. In the case of trastuzumab, the mechanisms responsible for its antitumor activity might be different in different clinical settings [4]. In general, long-term application of an antibody-based therapy is usually required to maintain the cytostatic function and to prevent tumor regrowth [4]. For many patients, this eventually triggers a clonal selection of malignant cells with alternative proliferation signaling pathways and leads to the development of resistance.

The addition of a cytotoxic payload to a mAb is a way to increase its potency. Such antibody-drug conjugates (ADCs) take advantage of the specific binding of mAbs to cell-surface antigens to selectively deliver the cytotoxic payload to the tumor cells [5,6]. However, this approach has a number of challenges. The cytostatic action of an antibody may arrest the cell cycle in the G1 phase, when cancer cells are less sensitive to the cytotoxic action of antimitotic agents, such as DM1 or MMAE [7]. Furthermore, resistance to the action of the payload may also develop over time by tumor cells [8,9,10]. It has also been shown that in some patients treated with trastuzumab, a selection of non-HER2-expressing malignant cells takes place [11]. In the case of the HER2-targeting ADC trastuzumab emtansine (T-DM1), low HER2 expression was suggested to be one of the reasons for resistance to treatment [10]. To achieve an effective intracellular concentration of DM1 in cancer cells with low HER2 expression, a more efficacious delivery of DM1 is required. However, for many solid tumors, the size of the mAb (150 kDa) hampers extravasation as well as penetration into the tumor’s interior [12]. Moreover, increasing the number of drugs per mAb molecule, the drug-to-antibody ratio (DAR), might alter the binding properties and physical stability of the ADC, as well as cause faster clearance through the liver due to an increased hydrophobicity [13,14].

It is relatively complex to engineer and produce ADCs. Most ADCs approved for clinical use utilize stochastic attachment of the payload to, e.g., lysines. Due to its large size, a mAb has dozens of potential positions for drug conjugation in those cases. This leads to complex mixtures of molecules with different DAR and positions of the payload. Administering of such mixtures could lead to sub-optimal pharmacokinetics, therapeutic efficacy and toxicity [14]. Despite the development of methods for site-specific conjugation, it is still challenging to achieve a homogenous compound with an optimal DAR [15].

One possible solution for tumor-specific drug delivery is the use of non-antibody targeting proteins. Unlike antibodies, engineered scaffold proteins (ESPs) are generally small (<20 kDa), which permits better extravasation and penetration into solid tumors [16]. Several classes of ESPs, e.g., affibody molecules, designed ankyrin repeat proteins (DARPins) and albumin-binding domain (ABD)-derived affinity proteins (ADAPTs), have previously been evaluated both for imaging and therapeutic purposes and have demonstrated promising tumor-targeting properties [17,18,19,20,21,22]. Unlike mAbs, ESPs can be produced in prokaryotic organisms or, the smallest ones, by peptide synthesis with high yield and at a low cost. Genetic engineering allows for facile modification of the scaffold and development of multispecific constructs, which enables the possibility to fine-tune its properties. Many ESPs also lack cysteines in the framework. This property can be capitalized upon, and cysteines can be introduced at desired positions for site-specific conjugation of the drugs, providing a conjugate with a well-controlled DAR [23]. The robustness, high solubility and thermodynamic stability of many ESPs makes them more tolerant to harsh conditions, which are sometimes encountered during conjugation and radionuclide labeling, in comparison to mAbs [24].

The feasibility of using ESPs for tumor-targeted delivery of drugs and toxins has been studied for affibody molecules [18,25,26,27], DARPins [20,28,29,30,31,32,33], adnectins [34,35] and anticalins [36]. The HER2-targeting ESPs described in the literature interacts with different epitopes on the receptor; e.g., the HER2-targeting affibody molecules bind to subdomain III, the DARPin 9_29 binds to subdomain I and the DARPin G3 binds to subdomain IV [37]. The differences in protein scaffold composition also lead to different off-target interactions, which in turn lead to differences in the uptake in normal organs and tissues in vivo. Expanding the targeting scaffold repertoire and investigating their structure–property relationship increases the chances to develop an optimal targeting agent for any given application.

We recently developed a HER2-targeting protein based on the ADAPT scaffold [22,38,39]. The ADAPT scaffold was developed from an albumin binding domain (ABD) from streptococcal protein G and is a small (5 kDa) three-helical bundle. Randomization of amino acids located in the helices one and three in ABD resulted in combinatorial libraries permitting selection of ADAPTs that could bind selectively to different targets while their binding to human serum albumin (HSA) by helix two was preserved [40]. This strategy was used to develop ADAPTs targeting tumor necrosis factor-α (TNF-α) [40], human epidermal growth factor receptor 3 (HER3) [41] and HER2 [42]. Later, the albumin-binding ability was removed to generate ADAPT6, targeting HER2 and having a short in vivo half-life [38]. ADAPT6 binds to the same epitope on HER2 as trastuzumab (subdomain IV) [37]. It has shown excellent tumor-targeting properties and has provided high imaging contrast in vivo, which enabled its clinical translation as a HER2-imaging probe. ADAPT6 labeled with technetium-99m was evaluated in a phase I clinical trial and was well tolerated by patients without any adverse reactions [21]. It provided excellent discrimination between HER2-positive and HER2-negative tumors in patients with primary breast cancer already at 2 h after injection. These results support the use of ADAPT6 as a promising protein scaffold for development of HER2-targeting therapeutics.

**Figure 1:**
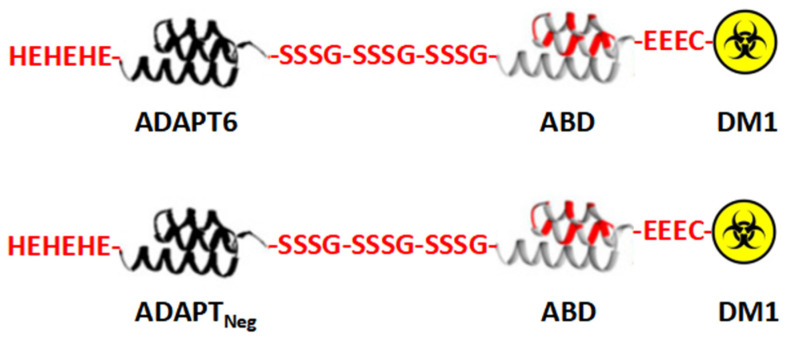
Schematic representation of the molecular design of the conjugates consisting of an albumin-binding domain (ABD) derived affinity protein (ADAPT6 or ADAPT_Neg_), a (SSSG)_3_ linker, an ABD and the cytotoxic drug DM1, used in this study.

In a previous study, an ADAPT6-based drug conjugate was designed for targeted delivery of a cytotoxic drug to HER2-expressing tumor cells [39]. The ADAPT6-ABD-mcDM1 conjugate consists of ADAPT6 as the HER2-targeting domain, an ABD for extension of the half-life in blood circulation for increased bioavailability, and the cytotoxic drug DM1 (Figure 1). ADAPT6-ABD-mcDM1 demonstrated nanomolar affinity to HER2, a reasonably good internalization rate by HER2-expressing cancer cells lines, a specific HER2-dependent toxicity in several cancer cell lines, and the capacity for specific accumulation in HER2-expressing human tumor xenografts in mice.

As mentioned earlier, one of the hurdles for cancer therapy is the clonal selection. In disseminated cancer with multiple metastatic sites, the HER2 expression level in the primary tumor and in the metastatic sites might be different [43]. Due to the heterogeneity of HER2 expression, it is challenging to determine its level in all metastatic sites using a conventional biopsy-based approach. Radionuclide molecular imaging might provide information about HER2 expression in all lesions and could be used to select patients with HER2-positive lesions for HER2-targeted therapy [44]. In addition, this method is non-invasive and can be performed repeatedly to monitor changes in HER2 expression over time. Several formats of imaging agents could be considered for imaging of HER2 during ADAPT6-based targeted therapy: ADAPT6-ABD-mcDM1 itself or smaller formats, such as ADAPT6 itself or an affibody molecule.

The primary goal of this study was to test the hypothesis that targeted therapy using ADAPT6-ABD-mcDM1 can significantly improve survival of mice bearing HER2-overexpressing ovarian cancer xenografts. The secondary goal was to evaluate several imaging probes (^99m^Tc-labeled ADAPT6-ABD-mcDM1, ^99m^Tc-labeled ADAPT6 and ^99m^Tc-labeled affibody molecules Z_HER2:41071_) for visualization of HER2 expression in tumors for potential stratification of patients for ADAPT6-ABD-mcDM1-based therapy and monitor their response to treatment.

## 2. Materials and Methods

### 2.1. General

The chemicals used in the study were purchased from Sigma-Aldrich (Sweden AB, Stockholm, Sweden). Buffers were prepared using high-quality Milli-Q water.

ADAPT6, ADAPT6-ABD-mcDM1 and ADAPT_Neg_-ABD-mcDM1 were produced and characterized as described previously [39]. The HER2-targeting affibody molecule Z_HER2:41071_ [17] was kindly provided by Affibody AB (Solna, Sweden).

### 2.2. Cell Culture

The human ovarian cancer cell line SKOV3 was purchased from American Type Culture Collection (ATCC via LGC Promochem, Borås, Sweden) and cultured in Roswell Park Memorial Institute (RPMI)-1640 medium supplemented with 10% fetal bovine serum (FBS), 2 mM L-glutamine, penicillin 100 IU/mL and 100 µg/mL streptomycin. The cells were grown in a humidified incubator at 37 °C and 5% CO_2_. Cells were detached using a trypsin-ethylenediaminetetraacetic acid (EDTA) solution (0.25% trypsin, 0.02% EDTA in buffer).

### 2.3. Experimental Therapy Study

The animal experiments were performed in agreement with national legislation on laboratory animals’ protection and were approved by the Ethics Committee for Animal Research in Uppsala (permit 5.8.18-11931/2020, approved 28 August 2020).

**Figure 2:**
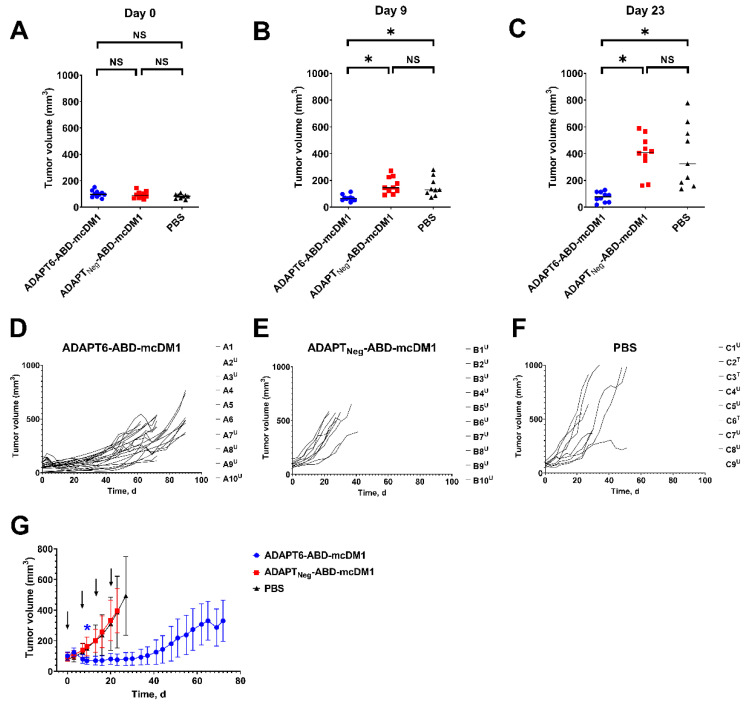
Comparison of tumor volumes in experimental therapy groups (n = 9–10) in BALB/c nu/nu mice bearing SKOV3 xenografts, receiving ADAPT6-ABD-mcDM1, ADAPT_Neg_-ABD-mcDM1 or PBS (vehicle) (**A**) at the start of the experiment (day 0), (**B**) after two treatment cycles (day 9) and (**C**) after four treatment cycles (day 23). Individual tumor volume growth curves for mice received (**D**) ADAPT6-ABD-mcDM1, (**E**) ADAPT_Neg_-ABD-mcDM1 or (**F**) PBS (vehicle). The mice were euthanized when the volume of the subcutaneous xenografts exceeded 1000 mm^3^ (T) or ulcers on the xenografts were observed (U). (**G**) The average tumor volume (n = 9–10) in mice receiving ADAPT6-ABD-mcDM1, ADAPT_Neg_-ABD-mcDM1 or PBS (vehicle) once a week for four weeks. The data are presented as an average value ± standard deviation (SD). The curves were drawn until 30–33% of the mice in a group were euthanized. The arrows indicate the days when treatment was administered. NS corresponds to no statistically significant difference (*p* > 0.05); * corresponds to *p* < 0.01 (one-way ANOVA with Bonferroni correction).

The SKOV3 xenografts were established in female BALB/c nu/nu mice by subcutaneous implantation of 1 × 10^7^ SKOV3 cells in 100 μL of medium in the abdominal region. The therapy started one week after the implantation. The mice were randomized in three groups (n = 9–10). One group of mice received intravenous (i.v.) injections of 13.3 mg/kg of ADAPT6-ABD-mcDM1 in 100 μL of PBS, the second group of mice received i.v. injections of the same dose of ADAPT_Neg_-ABD-mcDM1 in 100 μL of PBS, and the third group of mice received i.v. injections of 100 μL of PBS. The average tumor volume at the start of treatment was 101 ± 26, 94 ± 26 and 77 ± 22 mm^3^ for mice treated with ADAPT6-ABD-mcDM1, ADAPT_Neg_-ABD-mcDM1 and PBS, respectively. There were no significant differences between the tumor volumes in the different groups (Figure 2A). The average body weight was 18.8 ± 1.1 g. The injections were performed once a week for four consecutive weeks. Tumor volumes and body weights were measured twice a week. The tumors were measured using a digital caliper for the largest longitudinal (length) and transverse (width) diameter, and the tumor volumes were calculated using the formula: tumor volume = 1/2 (length × width^2^). The relative tumor volume (RTV) was calculated using the formula: RTV = (tumor volume on the day of measurement)/(tumor volume on day 0).

The mice were euthanized at predetermined humane end-points: tumor size exceeding 1000 mm^3^, tumor ulceration, internal bleeding, and total weight loss of 15% from the treatment initiation or 10% within one week. The study was terminated 90 days after the first injection according to requirements of the ethical permit. After the mice were sacrificed, kidneys and livers were fixed in 10% formalin solution for 24 h and stored in ethanol. The tissues were embedded in paraffin according to the standard operating procedure, sectioned (3–4 µm thickness), stained with hematoxylin-eosin (HE) and examined for histopathological changes. The pathology examination was performed by a veterinary pathologist at BioVet AB veterinary medicine laboratory (Sollentuna, Sweden).

### 2.4. Imaging during Experimental Therapy

The imaging experiment was performed to determine an optimal tracer for non-invasive visualization of HER2 in malignant tumors for potential stratification of patients and for monitoring response to therapy using ADAPT6-ABD-mcDM1.

Radiolabeling of ADAPT6-ABD-mcDM1 and ADAPT6 using [^99m^Tc(CO)_3_(H_2_O)_3_]^+^ (tricarbonyl technetium) precursor was performed as described earlier [39,45]. Radiolabeling of Z_HER2:41071_ with technetium-99m was performed as described earlier [17].

The radiochemical yield and purity were measured using iTLC silica gel strips (Varian, Lake Forest, CA, USA) in PBS. The distribution of activity along the strip was measured using a Storage Phosphor System (CR35 BIO Plus, ElysiaRaytest, Bietigheim-Bissingen, Germany) and analyzed with AIDA Image Analysis software (ElysiaRaytest, Bietigheim-Bissingen, Germany).

Imaging of HER2 expression was performed in two mice from each group using ^99m^Tc-Z_HER2:41071_. At day 29 (ADAPT_Neg_-ABD-mcDM1 group), day 34 (PBS group) and day 43 (ADAPT6-ABD-mcDM1 group), two mice were injected i.v. with ^99m^Tc-Z_HER2:41071_ (5 μg, 15–17 MBq in 100 μL of PBS), and whole body micro-single photon emission computed tomography (SPECT)/CT scans were performed at 4 h post-injection (p.i.) using a nanoScan SPECT/CT (Mediso Medical Imaging Systems, Budapest, Hungary) as described earlier [17]. The SPECT acquisition time was 10 min. The same mice from the ADAPT6-ABD-mcDM1 group were imaged again at day 77, using the same protocol.

To visualize the biodistribution of ADAPT6-ABD-mcDM1, at day 36 (two days after imaging with ^99m^Tc-Z_HER2:41071_) the same mice from the PBS group were imaged with ^99m^Tc(CO)_3_-ADAPT6-ABD-mcDM1 (6 μg, 16 MBq in 100 μL of PBS) at 4 h and 24 h p.i. The SPECT acquisition time was 10 min at 4 h p.i. and 60 min at 24 h p.i.

### 2.5. Statistics

The data were analyzed using GraphPad Prism (9.3.1 for Windows, GraphPad Software, La Jolla, CA, USA). One-way ANOVA with Bonferroni correction for multiple comparisons was used to compare the values between multiple groups and determine statistically significant differences (*p <* 0.05). The survival data were analyzed using a Mantel–Cox log-rank test. The therapy outcomes (exponential tumor growth, delayed exponential tumor growth, controlled tumor growth (tumor ulceration without tumor growth)) were analyzed by a χ-square test.

## 3. Results

### 3.1. Experimental Therapy

The therapeutic potency of ADAPT6-ABD-mcDM1 was investigated in mice bearing SKOV3 xenografts. There was no statistically significant (*p >* 0.05) difference in tumor volume between the groups at day 0 (Figure 2A). After two cycles of treatment, at day 9, the tumors in the ADAPT6-ABD-mcDM1 group (69 ± 23 mm^3^) were significantly (*p <* 0.01) smaller than tumors in the ADAPT_Neg_-ABD-mcDM1 group (163 ± 61 mm^3^) and the PBS group (152 ± 71 mm^3^) (Figure 2B). The average tumor size in the group treated with ADAPT6-ABD-mcDM1 remained significantly smaller compared to the other groups until day 23 (the last day when all mice in all groups were alive) (Figure 2C).

The individual tumor growth curves are shown in Figure 2D–F, the individual tumor volumes are shown in Table S1, the average tumor volumes are shown in Figure 2G, and the relative volumes are shown in Figure S1. The tumors in the ADAPT_Neg_-ABD-mcDM1 group (tumor doubling time 11.5 d, 95% CI from 10 to 13.5 d) and in the PBS group (tumor doubling time 12.1 d, 95% CI from 9.6 to 15.6 d) grew rapidly. The therapy outcome of the mice in the ADAPT_Neg_-ABD-mcDM1 group was that 100% mice were euthanized due to tumor ulceration. In the PBS group, six out of nine (67%) mice were euthanized due to tumor ulceration and three out of nine (33%) mice were euthanized when tumors reached the size limit. The tumor growth in the ADAPT6-ABD-mcDM1 group was inhibited until day 40, followed by a slow regrowth of all tumors (tumor doubling time 31 d, 95% CI from 21 to 55 d). The therapy outcome was that six out of ten (60%) mice were euthanized due to tumor ulceration. Four out of ten (40%) mice had controlled tumor growth and survived until the study termination (day 90), which was the maximum study length according to the ethical permit.

**Figure 3:**
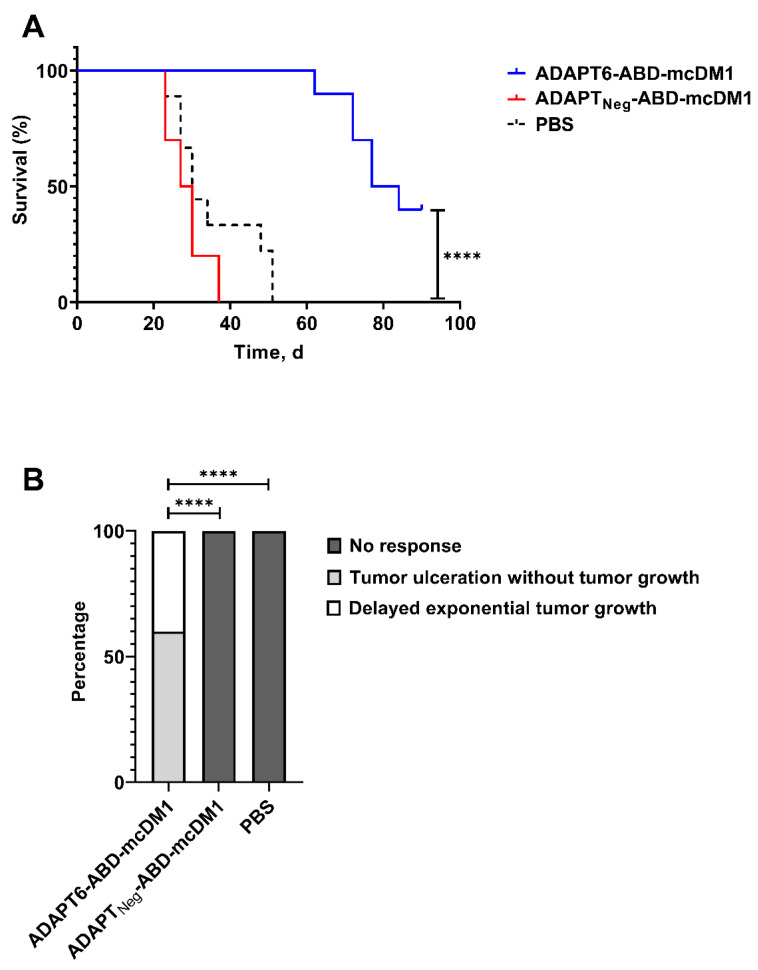
(**A**) Survival of BALB/c nu/nu mice bearing SKOV3 xenografts treated with ADAPT6-ABD-mcDM1, ADAPT_Neg_-ABD-mcDM1 or PBS (vehicle). (**B**) Therapy outcomes for different treatment groups. The output categories were defined as no response (exponential tumor growth), delayed exponential tumor growth and controlled tumor growth (tumor ulceration without tumor growth). The difference between groups was determined using chi–square test. **** corresponds to *p* < 0.0001.

The median survival was significantly (Mantel–Cox log-rank test, *p* < 0.0001) longer in the ADAPT6-ABD-mcDM1 group (80.5 days) compared to the ADAPT_Neg_-ABD-mcDM1 group (28.5 days) and to the PBS group (30 days) (Figure 3A). The therapy outcome was significantly (chi–square test, *p* < 0.0001) better for the ADAPT6-ABD-mcDM1 group than for the ADAPT_Neg_-ABD-mcDM1 group or the PBS group (Figure 3B).

**Figure 4:**
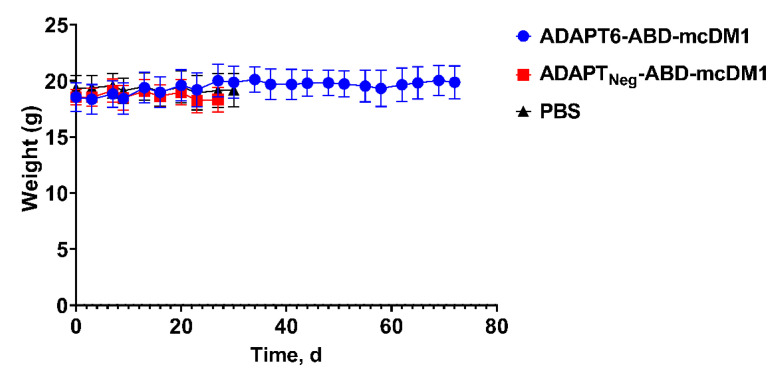
Average animal weight in each group during the therapy experiment. The data are presented as an average value ± SD. The curves were drawn until 30–33% of the mice in a group were euthanized.

The treatment was well tolerated, with no observable side effects. No tendency for weight loss was observed in the ADAPT6-ABD-mcDM1 group compared to the ADAPT_Neg_-ABD-mcDM1 group or the PBS group. The differences in average animal weight between the groups were within one standard deviation (Figure 4).

The histopathological examination results are shown in Figures S2 and S3. In the examined material, no lesions that would suggest toxicity of the treatment regimens were found in the liver or kidneys.

### 3.2. HER2 Imaging

The radiochemical yields of ^99m^Tc(CO)_3_-ADAPT6-ABD-mcDM1 and ^99m^Tc(CO)_3_-ADAPT6 were 87% and 98%, respectively, and the purity after size-exclusion purification was close to 100% for both compounds. The radiolabeling of ^99m^Tc-Z_HER2:41071_ provided a radiochemical yield of 98 ± 2% (n = 3). No purification of this compound was performed.

**Figure 5:**
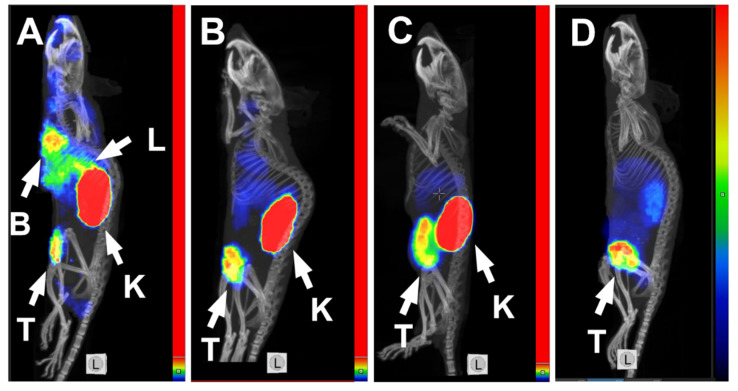
MicroSPECT/CT imaging of HER2-expression in mice from the PBS group using ^99m^Tc(CO)_3_-ADAPT6-ABD-mcDM1 at (**A**) 4 h and (**B**) 24 h p.i., (**C**) using ^99m^Tc(CO)_3_-ADAPT6 at 4 h p.i. and (**D**) using ^99m^Tc-Z_HER2:41071_ at 4 h p.i. The arrows with the letters “T”, “K”, “L” and “B” point to the tumors, kidneys, liver and blood pool in the heart, respectively. The color scale sidebars show the relative activity and are adjusted to the first red pixel in tumors.

^99m^Tc(CO)_3_-ADAPT6-ABD-mcDM1 provided a clear visualization of HER2-expressing SKOV3 xenografts using SPECT/CT already at 4 h p.i. (Figure 5A). Besides the tumor, a high accumulation of the labeled compound was visualized in kidneys, and activity accumulation in the blood pool in heart and liver was comparable with the accumulation in the tumor. By 24 h p.i. (Figure 5B), the activity was cleared to a high extent from blood and liver, with kidneys and tumor being the only sites with a prominent activity accumulation. Imaging using ^99m^Tc(CO)_3_-ADAPT6 also provided a clear visualization of the tumor at 4 h p.i. but with a substantial accumulation of activity in kidneys. In comparison, ^99m^Tc-Z_HER2:41071_ at 4 h p.i. provided an appreciably higher uptake in the SKOV3 xenografts than in any other tissue, enabling high-contrast visualization of the tumors (Figure 5C).

**Figure 6:**
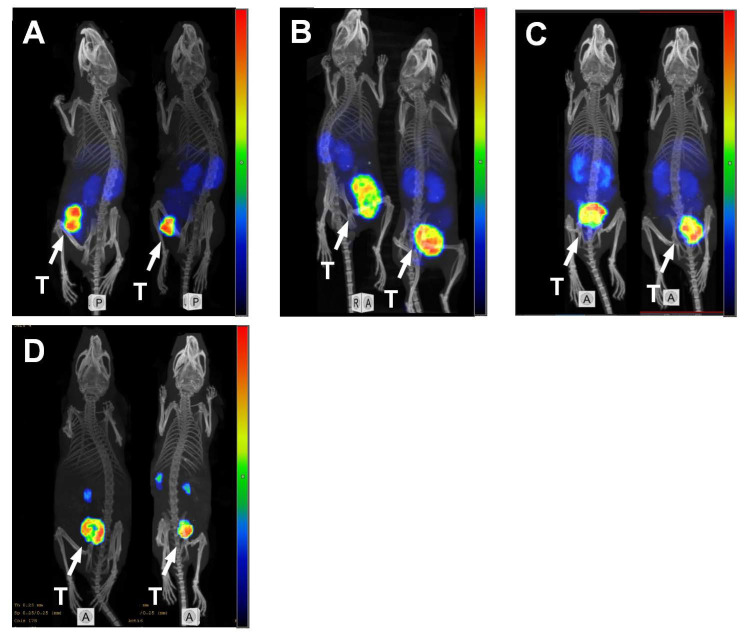
MicroSPECT/CT imaging of HER2-expression using ^99m^Tc-Z_HER2:41071_ in BALB/c nu/nu mice bearing SKOV3 xenografts. The imaging was performed at days 43 (**A**), 29 (**B**), 34 (**C**) and 77 (**D**) of the experimental therapy. Two mice from the ADAPT6-ABD-mcDM1 treated group with small tumors at day 43 (**A**) and day 77 (**D**), two mice from ADAPT_Neg_-ABD-mcDM1 treated group at day 29 (**B**) and two mice from PBS treated group at day 34 (**C**) with large tumors. The arrow with the letter “T” points to the tumors.

SPECT/CT imaging of HER2 expression using ^99m^Tc-Z_HER2:41071_ was performed in mice during therapy (Figure 6). The imaging enabled a clear visualization of the tumors in all cases. The tumor burden observed on imaging correlated well with the caliper measurements of tumor size (Table S1).

## 4. Discussion

The development of ADCs remains to be a mainstream direction in the field of targeted drug delivery. Currently, fourteen ADCs have been approved for clinical use, and over 100 are in clinical trials [46]. Despite this great progress, ADCs still face many challenges, such as complexity of development, production and quality control, variability in pharmacokinetics, toxicities due to payload release in blood circulation, insufficient tumor targeting, as well as drug resistance.

Alternative targeting proteins based on non-immunoglobulin scaffolds, such as affibody molecules, DARPins, ADAPTs and adnectins, have the advantage of facile genetic engineering to include additional domains, e.g., an ABD for extension of the half-life in blood, and sites for precise modification, e.g., attachment of drugs, providing uniform drug conjugates with well-defined properties. Due to more favorable structural characteristics, ESPs have higher tolerance to pH and temperature changes than mAbs, resulting in better in vitro and in vivo stability of the drug conjugates. Several conjugates of affibody molecules [18,25,26], DARPins [20,31,32,33] and adnectins [35] with drugs and toxins have been developed and have demonstrated anti-tumor activity in preclinical studies.

The current study enhances this armamentarium by investigating a drug conjugate based on the ADAPT scaffold protein. Fusion of the HER2-targeting ADAPT6 to ABD_035_ with femtomolar affinity to human serum albumin was used to prolong the conjugate’s half-life in circulation. Binding to albumin increases the molecular weight of the conjugate from 14 to 81 kDa, which prevents excretion via glomerular filtration in kidneys. In addition, the half-life extension by the ABD is likely mediated by indirect targeting of the neonatal Fc receptor (FcRn) [47]. We previously found that the ABD placement at C-terminus of ADAPT6 provided the most optimal pharmacokinetics and tumor-targeting properties [22]. The maytansine derivative DM1, commonly used in ADCs, was conjugated to the C-terminal cysteine residue via a non-cleavable maleimidocaproyl (mc) linker. The ADAPT6-ABD-mcDM1 drug conjugate has been extensively characterized in vitro, and its biodistribution and tumor-targeting has been evaluated in vivo [39].

In this study, we investigated the therapeutic efficacy of ADAPT6-ABD-mcDM1 in mice bearing HER2-expressing SKOV3 ovarian cancer xenografts and compared it with a non-targeting homologous construct (ADAPT_Neg_-ABD-mcDM1) and a vehicle control. The treatment with ADAPT6-ABD-mcDM1 resulted in a significant delay of tumor growth in comparison with both the non-targeting and the vehicle-treated control groups (Figure 2, Table S1). The therapeutic effect was apparently caused by targeted delivery of DM1 and not by anti-proliferative effect due to the presence of the conjugate in blood circulation or its accumulation in xenografts due to the enhanced permeability and retention (EPR) effect, as the homologous ADAPT_Neg_-ABD-mcDM1 did not inhibit tumor growth. This resulted in a significantly improved median survival of mice treated with ADAPT6-ABD-mcDM1 (Figure 3A). The growth of all tumors in mice treated using ADAPT6-ABD-mcDM1 was well controlled until ca. day 40, after which it increased exponentially in 40% of mice. In all mice treated using ADAPT_Neg_-ABD-mcDM1 or vehicle, the tumors grew rapidly and followed an exponential growth trajectory (Figure 2E and Figure 3B). However, despite the delayed tumor growth caused by treatment with ADAPT6-ABD-mcDM1 at a 13.3 mg/kg dose, only 40% of mice survived until the end of the study while having a significant tumor burden (tumor volume over 370 mm^3^). In comparison with a previous study using an affibody-based Z_HER2_-ABD-mcDM1 conjugate at a lower dose of (10.3 mg/kg), where at the study end-point 70% of mice had tumors below 50 mm^3^ and 30% of mice had tumor below 200 m^3^, the therapeutic effect of the ADAPT6-based drug conjugate was not as pronounced [18]. This indicates that a higher dose of ADAPT6-ABD-mcDM1 is needed to achieve the same therapeutic effect as for Z_HER2_-ABD-mcDM1.

During the development and characterization of ADAPT6-ABD-mcDM1, one of the concerns was an elevated renal uptake, which appreciably exceeded the uptake in the tumor. The injected dose of ADAPT6-ABD-mcDM1 (13.3 mg/kg) in the current study was lower than the highest dose of the affibody-drug conjugate Z_HER2_-ABD-mcDM1 (15.1 mg/kg) used in a previous study [18]. Injection of 15.1 mg/kg of Z_HER2_-ABD-mcDM1 caused weight loss for some animals; however, the histopathology investigation did not reveal any pathological changes in kidneys or livers. Compared to Z_HER2_-ABD-mcDM1, ADAPT6-ABD-mcDM1 had lower blood retention, higher kidney uptake and equal liver uptake. The tumor uptake of ADAPT6-ABD-mcDM1 was lower at 24 and 48 h p.i. [18,39]. This data suggested that the therapy using ADAPT6-ABD-mcDM1 might have a higher risk of hepatic and renal toxicities with lower anti-tumor effect. Still, the histology evaluation of kidneys and livers from mice treated with ADAPT6-ABD-mcDM1 did not reveal any pathological changes (Figures S2 and S3). One of the possible explanations for the pronounced anti-tumor effect and the absence of toxicities to liver and kidneys despite higher uptake of ADAPT6-ABD-mcDM1 in these organs could be the preferential cytotoxicity of DM1 to rapidly dividing cancer cells in a tumor in comparison to normal cells in liver and kidneys with lower proliferation rate. This suggests that a maytansine derivative DM1 or the auristatin derivatives MMAE and MMAF, with similar mechanisms of action, offer an additional advantage of lower general toxicity, in comparison with, e.g., bacterial toxins, which are cytotoxic to all types of cells.

Experience with other scaffold proteins suggests that the biodistribution profile of ADAPT6-based drug conjugate might be improved by optimizing the molecular design of the construct [48] as well as the linkers between the domains [49] and between the protein and cytotoxic moieties [31,50]. This could further reduce the toxicity to normal organs and increase the therapeutic window. This study has provided a proof-of-principle for a targeted delivery of non-radioactive cytotoxic payload to tumors using the ADAPT scaffold. Further studies should include an optimization of format and payload. Such work could be promoted by the use of a radioactive label to find variants providing the highest accumulations in tumors and the lowest in normal tissues. The anti-tumor activity of the best variant should be verified using other HER2-expressing ovarian cancer cell lines, such as, e.g., OVCAR-2 and OVCAR-3. The possible impact of HER2 expression level on the antitumor effect should be also evaluated before proceeding to clinical development.

Another approach to further reduce the toxicity could be the conjugation of ADAPT6 with another cytotoxic payload having a different toxicity profile [20,51]. In our previous study, ADAPT6-ABD was conjugated with the bacterial toxin derivative PE38X8 [52]. ADAPT6-ABD-PE38X8 had two-to-three–fold lower kidney uptake (58.0 ± 3.0 %ID/g at 4 h p.i. and 46.0 ± 2.0 %ID/g at 24 h p.i.) than ADAPT6-ABD-mcDM1 (163.6 ± 14.7 %ID/g at 4 h p.i. and 82.7 ± 3.5 %ID/g at 24 h p.i.), indicating lower renal toxicity risk, but higher liver uptake (9.0 ± 0.4 %ID/g at 4 h p.i. and 6.0 ± 1.0 %ID/g at 24 h p.i.) than ADAPT6-ABD-mcDM1 (5.96 ± 1.07 %ID/g at 4 h p.i. and 4.83 ± 0.27 %ID/g at 24 h p.i.), indicating higher hepatic toxicity risk [39,52].

The imaging of HER2 using the radiolabeled drug conjugate ^99m^Tc(CO)_3_-ADAPT6-ABD-mcDM1 provides the advantages of both visualizing HER2 expression in tumors and reporting the receptor accessibility by the targeted drug. Despite good tumor targeting and accumulation of ^99m^Tc(CO)_3_-ADAPT6-ABD-mcDM1 in the tumor, a high accumulation of activity in liver was also observed (Figure 5A). The liver is a common metastatic site for ovarian and breast cancers; thus, high liver background might reduce the sensitivity of HER2 imaging in this metastatic site. Imaging at a later time point (24 h p.i.) improved the tumor-to-liver contrast due to a reduction in liver uptake (Figure 5B). However, to achieve an appropriate image contrast using ^99m^Tc(CO)_3_-ADAPT6-ABD-mcDM1 would require a 24 h delay between the injection and imaging, higher injected activity and higher dose burden for patients.

In contrast, the non-ABD fused ADAPT6 labeled with ^99m^Tc(CO)_3_ provided a comparable imaging contrast at the day of injection, with good correlation between imaging results and therapy outcomes (Figure 5C). The smaller affibody-based imaging probe, Z_HER2:41071_, labeled with technetium-99m showed much lower accumulation of activity in normal organs, including liver and kidneys, which resulted in a high-contrast image at 4 h p.i. (Figure 5D). Dosimetry evaluation showed that for both ^99m^Tc(CO)_3_-ADAPT6 and ^99m^Tc-Z_HER2:41071_, very low effective doses were reached, with the lowest dose being for ^99m^Tc-Z_HER2:41071_ (0.00066 mSv/MBq) [17] and a medium dose for ^99m^Tc(CO)_3_-ADAPT6 (0.010 ± 0.003 mSv/MBq) [21], in comparison to the doses of clinically used imaging probes, such as ^111^In-trastuzumab (0.19 ± 0.02 mSv/MBq) [53] and ^111^In-pertuzumab (0.05 mSv/MBq) [54]. Another advantage of using an anti-HER2 affibody molecule as the imaging agent is that it does not compete with ADAPT6 for the binding to HER2 [38], which means that the imaging using ^99m^Tc-Z_HER2:41071_ could not only be used to select patients with HER2-positve lesions but could also be performed repeatedly during and after the treatment to monitor the therapeutic effect and disease progression, as we demonstrated in Figure 6.

## 5. Conclusions

Treatment using the ABD-fused DM1-conjugate based on the ADAPT6 scaffold protein increased survival of mice bearing HER2-expressing ovarian cancer xenografts without inducing toxicity to liver and kidneys. Imaging using ^99m^Tc(CO)_3_-ADAPT6-ABD-mcDM1 might provide information about the accessibility of HER2 in tumors for the therapeutic counterpart, while small non-ABD-fused radiolabeled scaffold proteins, such as ^99m^Tc-Z_HER2:41071_ and ^99m^Tc(CO)_3_-ADAPT6, might be better diagnostic companions for stratification of patients and monitoring of response to ADAPT6-ABD-mcDM1-based therapy.

## Data Availability

The data generated during the current study are available from the corresponding authors upon reasonable request.

## Supplementary Materials

The following supporting information can be downloaded at: https://www.mdpi.com/article/10.3390/pharmaceutics14081612/s1: Figure S1, Relative tumor growth curve of BALB/C nu/nu mice bearing SKOV3 xenografts; Figure S2, Results of pathological examination of kidneys; Figure S3, Results of pathological examination of livers; Table S1, Tumor volume (mm^3^) of ADAPT6-ABD-mcDM1 therapy experiments. Group A: ADAPT6-ABD-mcDM1; Group B: ADAPTNeg-ABD-mcDM1; Group C: PBS.

## References

[1] Janjigian Y.Y., Maron S.B., Chatila W.K., Millang B., Chavan S.S., Alterman C., Chou J.F., Segal M.F., Simmons M.Z., Momtaz P. (2020). First-line pembrolizumab and trastuzumab in HER2-positive oesophageal, gastric, or gastro-oesophageal junction cancer: An open-label, single-arm, phase 2 trial. Lancet Oncol..

[2] Karapetis C.S., Khambata-Ford S., Jonker D.J., O’Callaghan C.J., Tu D., Tebbutt N.C., Simes R.J., Chalchal H., Shapiro J.D., Robitaille S. (2008). K-ras mutations and benefit from cetuximab in advanced colorectal cancer. N. Engl. J. Med..

[3] Messersmith W.A., Ahnen D.J. (2008). Targeting EGFR in colorectal cancer. N. Engl. J. Med..

[4] Tagliabue E., Campiglio M., Pupa S.M., Ménard S., Balsari A. (2012). Activity and resistance of trastuzumab according to different clinical settings. Cancer Treat. Rev..

[5] Parakh S., Parslow A.C., Gan H.K., Scott A.M. (2016). Antibody-Mediated Delivery of Therapeutics for Cancer Therapy. Expert Opin. Drug Deliv..

[6] Costa R.L.B., Czerniecki B.J. (2020). Clinical Development of Immunotherapies for HER2 + Breast Cancer: A Review of HER2-Directed Monoclonal Antibodies and Beyond. NPJ Breast Cancer.

[7] Valabrega G., Montemurro F., Aglietta M. (2007). Trastuzumab: Mechanism of action, resistance and future perspectives in HER2-overexpressing breast cancer. Ann. Oncol..

[8] Barok M., Joensuu H., Isola J. (2014). Trastuzumab Emtansine: Mechanisms of Action and Drug Resistance. Breast Cancer Res..

[9] Hunter F.W., Barker H.R., Lipert B., Rothé F., Gebhart G., Piccart-Gebhart M.J., Sotiriou C., Jamieson S. (2020). Mechanisms of resistance to trastuzumab emtansine (T-DM1) in HER2-positive breast cancer. Br. J. Cancer.

[10] Shefet-Carasso L., Benhar I. (2015). Antibody-targeted drugs and drug resistance—Challenges and solutions. Drug Resist. Updat..

[11] Mittendorf E.A., Wu Y., Scaltriti M., Meric-Bernstam F., Hunt K.K., Dawood S., Esteva F.J., Buzdar A.U., Chen H., Eksambi S. (2009). Loss of HER2 amplification following trastuzumab-based neoadjuvant systemic therapy and survival outcomes. Clin. Cancer Res..

[12] Chauhan V.P., Stylianopoulos T., Boucher Y., Jain R.K. (2011). Delivery of molecular and nanoscale medicine to tumors: Transport barriers and strategies. Annu. Rev. Chem. Biomol. Eng..

[13] Sun X., Ponte J.F., Yoder N.C., Laleau R., Coccia J., Lanieri L., Qiu Q., Wu R., Hong E., Bogalhas M. (2017). Effects of drug-antibody ratio on pharmacokinetics, biodistribution, efficacy, and tolerability of antibody-maytansinoid conjugates. Bioconjug. Chem..

[14] Adem Y.T., Schwarz K.A., Duenas E., Patapoff T.W., Galush W.J., Esue O. (2014). Auristatin antibody drug conjugate physical instability and the role of drug payload. Bioconjug. Chem..

[15] Panowski S., Bhakta S., Raab H., Polakis P., Junutula J.R. (2014). Site-specific antibody drug conjugates for cancer therapy. mAbs.

[16] Wittrup K.D., Thurber G.M., Schmidt M.M., Rhoden J.J. (2012). Practical theoretic guidance for the design of tumor-targeting agents. Methods Enzymol..

[17] Oroujeni M., Rinne S.S., Vorobyeva A., Loftenius A., Feldwisch J., Jonasson P., Chernov V., Orlova A., Frejd F.Y., Tolmachev V. (2021). Preclinical Evaluation of 99mTc-ZHER2:41071, a Second-Generation Affibody-Based HER2-Visualizing Imaging Probe with a Low Renal Uptake. Int. J. Mol. Sci..

[18] Xu T., Ding H., Vorobyeva A., Oroujeni M., Orlova A., Tolmachev V., Gräslund T. (2021). Drug Conjugates Based on a Monovalent Affibody Targeting Vector Can Efficiently Eradicate HER2 Positive Human Tumors in an Experimental Mouse Model. Cancers.

[19] Bragina O., Chernov V., Schulga A., Konovalova E., Garbukov E., Vorobyeva A., Orlova A., Tashireva L., Sörensen J., Zelchan R. (2022). Phase I Trial of 99mTc-(HE)3-G3, a DARPin-Based Probe for Imaging of HER2 Expression in Breast Cancer. J. Nucl. Med..

[20] Xu T., Vorobyeva A., Schulga A., Konovalova E., Vorontsova O., Ding H., Gräslund T., Tashireva L.A., Orlova A., Tolmachev V. (2021). Imaging-Guided Therapy Simultaneously Targeting HER2 and EpCAM with Trastuzumab and EpCAM-Directed Toxin Provides Additive Effect in Ovarian Cancer Model. Cancers.

[21] Bragina O., von Witting E., Garousi J., Zelchan R., Sandström M., Orlova A., Medvedeva A., Doroshenko A., Vorobyeva A., Lindbo S. (2021). Phase I Study of 99mTc-ADAPT6, a Scaffold Protein–Based Probe for Visualization of HER2 Expression in Breast Cancer. J. Nucl. Med..

[22] Garousi J., von Witting E., Borin J., Vorobyeva A., Altai M., Vorontsova O., Konijnenberg M.W., Oroujeni M., Orlova A., Tolmachev V. (2021). Radionuclide Therapy Using ABD-Fused ADAPT Scaffold Protein: Proof of Principle. Biomaterials.

[23] Ding H., Xu T., Zhang J., Tolmachev V., Oroujeni M., Orlova A., Gräslund T., Vorobyeva A. (2021). Affibody-Derived Drug Conjugates Targeting HER2: Effect of Drug Load on Cytotoxicity and Biodistribution. Pharmaceutics.

[24] Gebauer M., Skerra A. (2009). Engineered protein scaffolds as next-generation antibody therapeutics. Curr. Opin. Chem. Biol..

[25] Altai M., Liu H., Ding H., Mitran B., Edqvist P.-H., Tolmachev V., Orlova A., Gräslund T. (2018). Affibody-Derived Drug Conjugates: Potent Cytotoxic Molecules for Treatment of HER2 over-Expressing Tumors. J. Control. Release.

[26] Zielinski R., Lyakhov I., Jacobs A., Chertov O., Kramer-Marek G., Francella N., Stephen A., Fisher R., Blumenthal R., Capala J. (2009). Affitoxin—A novel recombinant, HER2-specific, anticancer agent for targeted therapy of HER2-positive tumors. J. Immunother..

[27] Sochaj-Gregorczyk A.M., Serwotka-Suszczak A.M., Otlewski J.A. (2016). Novel Affibody-Auristatin E Conjugate with a Potent and Selective Activity Against HER2+ Cell Lines. J. Immunother..

[28] Sokolova E., Proshkina G., Kutova O., Shilova O., Ryabova A., Schulga A., Stremovskiy O., Zdobnova T., Balalaeva I., Deyev S. (2016). Recombinant targeted toxin based on HER2-specific DARPin possesses a strong selective cytotoxic effect in vitro and a potent antitumor activity in vivo. J. Control. Release.

[29] Sokolova E.A., Shilova O.N., Kiseleva D.V., Schulga A.A., Balalaeva I.V., Deyev S.M. (2019). HER2-Specific Targeted Toxin DARPin-LoPE: Immunogenicity and Antitumor Effect on Intraperitoneal Ovarian Cancer Xenograft Model. Int. J. Mol. Sci..

[30] Plückthun A. (2015). Designed ankyrin repeat proteins (DARPins): Binding proteins for research, diagnostics, and therapy. Annu. Rev. Pharmacol. Toxicol..

[31] Brandl F., Merten H., Zimmermann M., Béhé M., Zangemeister-Wittke U., Plückthun A. (2019). Influence of size and charge of unstructured polypeptides on pharmacokinetics and biodistribution of targeted fusion proteins. J. Control. Release.

[32] Karsten L., Janson N., Le Joncour V., Alam S., Müller B., Tanjore Ramanathan J., Laakkonen P., Sewald N., Müller K.M. (2022). Bivalent EGFR-Targeting DARPin-MMAE Conjugates. Int. J. Mol. Sci..

[33] Xu T., Liu Y., Schulga A., Konovalova E., Deyev S.M., Tolmachev V., Vorobyeva A. (2022). Epithelial cell adhesion molecule-targeting designed ankyrin repeat protein-toxin fusion Ec1-LoPE exhibits potent cytotoxic action in prostate cancer cells. Oncol. Rep..

[34] Hantschel O. (2017). Monobodies as possible next-generation protein therapeutics—A perspective. Swiss Med. Wkly..

[35] Lipovšek D., Carvajal I., Allentoff A.J., Barros A., Brailsford J., Cong Q., Cotter P., Gangwar S., Hollander C., Lafont V. (2018). Adnectin-drug conjugates for Glypican-3-specific delivery of a cytotoxic payload to tumors. Protein Eng. Des. Sel..

[36] Rothe C., Skerra A. (2018). Anticalin^®^ Proteins as Therapeutic Agents in Human Diseases. BioDrugs.

[37] Jost C., Schilling J., Tamaskovic R., Schwill M., Honegger A., Plückthun A. (2013). Structural basis for eliciting a cytotoxic effect in HER2-overexpressing cancer cells via binding to the extracellular domain of HER2. Structure.

[38] Garousi J., Lindbo S., Nilvebrant J., Åstrand M., Buijs J., Sandström M., Honarvar H., Orlova A., Tolmachev V., Hober S. (2015). ADAPT, a Novel Scaffold Protein-Based Probe for Radionuclide Imaging of Molecular Targets That Are Expressed in Disseminated Cancers. Cancer Res..

[39] Garousi J., Ding H., von Witting E., Xu T., Vorobyeva A., Oroujeni M., Orlova A., Hober S., Gräslund T., Tolmachev V. (2021). Targeting HER2 Expressing Tumors with a Potent Drug Conjugate Based on an Albumin Binding Domain-Derived Affinity Protein. Pharmaceutics.

[40] Nilvebrant J., Alm T., Hober S., Löfblom J. (2011). Engineering Bispecificity into a Single Albumin-Binding Domain. PLoS ONE.

[41] Nilvebrant J., Astrand M., Löfblom J., Hober S. (2013). Development and characterization of small bispecific albumin-binding domains with high affinity for ErbB3. Cell Mol. Life Sci..

[42] Nilvebrant J., Åstrand M., Georgieva-Kotseva M., Björnmalm M., Löfblom J., Hober S. (2014). Engineering of Bispecific Affinity Proteins with High Affinity for ERBB2 and Adaptable Binding to Albumin. PLoS ONE.

[43] Filho O.M., Viale G., Stein S., Trippa L., Yardley D.A., Mayer I.A., Abramson V.G., Arteaga C.L., Spring L.M., Waks A.G. (2021). Impact of HER2 Heterogeneity on Treatment Response of Early-Stage HER2-Positive Breast Cancer: Phase II Neoadjuvant Clinical Trial of T-DM1 Combined with Pertuzumab. Cancer Discov..

[44] Tolmachev V., Orlova A., Sörensen J. (2021). The emerging role of radionuclide molecular imaging of HER2 expression in breast cancer. Semin. Cancer Biol..

[45] Lindbo S., Garousi J., Åstrand M., Honarvar H., Orlova A., Hober S., Tolmachev V. (2016). Influence of Histidine-Containing Tags on the Biodistribution of ADAPT Scaffold Proteins. Bioconjug. Chem..

[46] Fu Z., Li S., Han S., Shi C., Zhang Y. (2022). Antibody drug conjugate: The “biological missile” for targeted cancer therapy. Signal Transduct. Target. Ther..

[47] Andersen J.T., Pehrson R., Tolmachev V., Daba M.B., Abrahmsén L., Ekblad C. (2011). Extending half-life by indirect targeting of the neonatal Fc receptor (FcRn) using a minimal albumin binding domain. J. Biol. Chem..

[48] Yin W., Xu T., Altai M., Oroujeni M., Zhang J., Vorobyeva A., Vorontsova O., Vtorushin S.V., Tolmachev V., Gräslund T. (2021). The Influence of Domain Permutations of an Albumin-Binding Domain-Fused HER2-Targeting Affibody-Based Drug Conjugate on Tumor Cell Proliferation and Therapy Efficacy. Pharmaceutics.

[49] Xu T., Zhang J., Oroujeni M., Tretyakova M.S., Bodenko V., Belousov M.V., Orlova A., Tolmachev V., Vorobyeva A., Gräslund T. (2022). Effect of Inter-Domain Linker Composition on Biodistribution of ABD-Fused Affibody-Drug Conjugates Targeting HER2. Pharmaceutics.

[50] Ding H., Altai M., Rinne S.S., Vorobyeva A., Tolmachev V., Gräslund T., Orlova A. (2019). Incorporation of a Hydrophilic Spacer Reduces Hepatic Uptake of HER2-Targeting Affibody–DM1 Drug Conjugates. Cancers.

[51] Shramova E., Proshkina G., Shipunova V., Ryabova A., Kamyshinsky R., Konevega A., Schulga A., Konovalova E., Telegin G., Deyev S. (2020). Dual Targeting of Cancer Cells with DARPin-Based Toxins for Overcoming Tumor Escape. Cancers.

[52] Liu H., Lindbo S., Ding H., Altai M., Garousi J., Orlova A., Tolmachev V., Hober S., Gräslund T. (2019). Potent and specific fusion toxins consisting of a HER2-binding, ABD-derived affinity protein, fused to truncated versions of Pseudomonas exotoxin A. Int. J. Oncol..

[53] Gaykema S.B., de Jong J.R., Perik P.J., Brouwers A.H., Schröder C.P., Munnink T.H.O., Bongaerts A.H., de Vries E.G., Hooge M.N.L.D. (2014). (111)In-trastuzumab scintigraphy in HER2-positive metastatic breast cancer patients remains feasible during trastuzumab treatment. Mol. Imaging.

[54] Lam K., Chan C., Done S.J., Levine M.N., Reilly R.M. (2015). Preclinical pharmacokinetics, biodistribution, radiation dosimetry and acute toxicity studies required for regulatory approval of a Clinical Trial Application for a Phase I/II clinical trial of (111)In-BzDTPA-pertuzumab. Nucl. Med. Biol..

