# Supplementary material for: Experimental HER2-Targeted Therapy Using ADAPT6-ABD-mcDM1 in Mice Bearing SKOV3 Ovarian Cancer Xenografts: Efficacy and Selection of Companion Imaging Counterpart
Source: Pharmaceutics. 2022 Aug 2;14(8):1612. doi: 10.3390/pharmaceutics14081612 (PMC9415843; doi:10.3390/pharmaceutics14081612)
Supplement: Supplementary file 1 [file pharmaceutics-14-01612-s001.zip › pharmaceutics-1825638-supplementary.pdf]

Supplementary Materials: Experimental HER2-targeted therapy using ADAPT6-ABD-mcDM1 in mice bearing SKOV3 ovarian cancer xenografts: efficacy and selection of companion imaging counterpart

Javad Garousi, Tianqi Xu, Yongsheng Liu, Olga Vorontsova, Sophia Hober, Anna Orlova, Vladimir Tolmachev, Torbjörn Gräslund, Anzhelika Vorobyeva

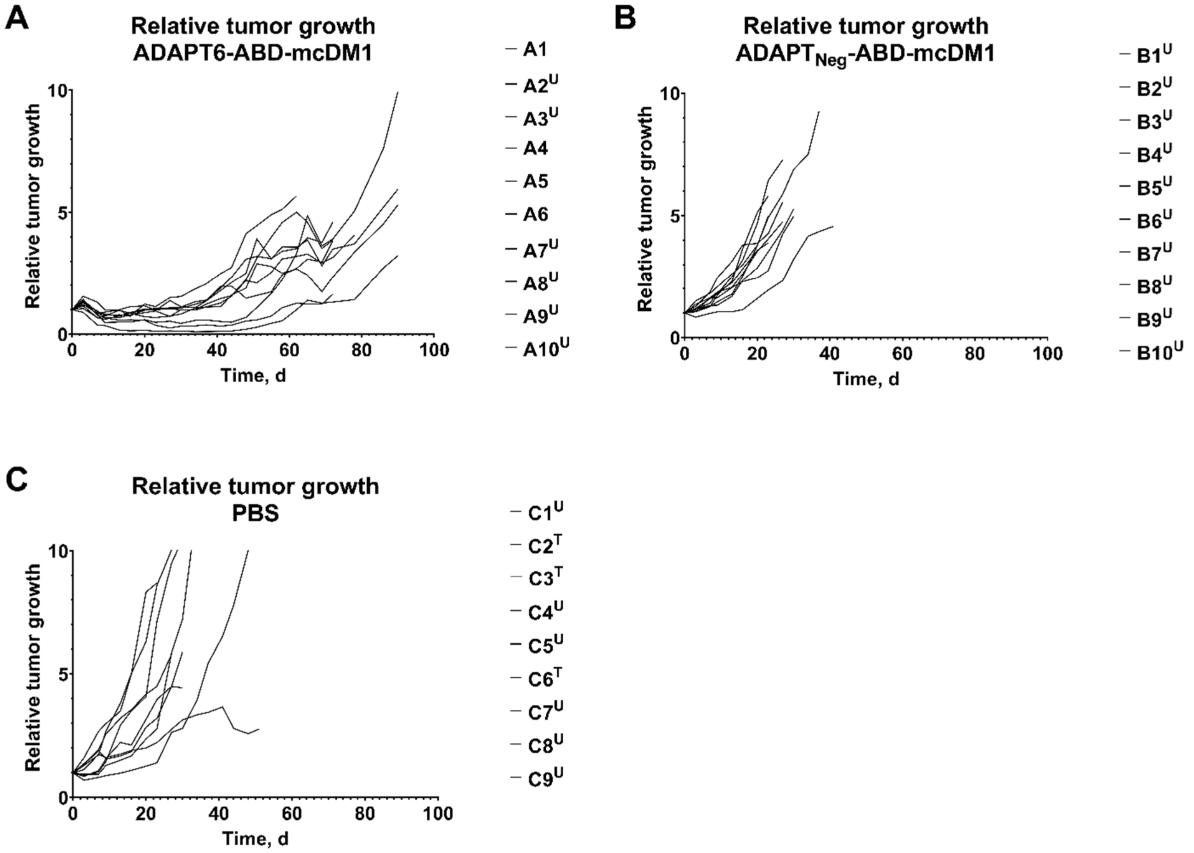

Figure S1. Relative tumor growth curve of BALB/C nu/nu mice bearing SKOV3 xenografts. Mice were treated with (**A**) ADAPT6-ABD-mcDM1, (**B**) ADAPTNeg-ABD-mcDM1 and (**C**) PBS. The mice were euthanized when the volume of the subcutaneous xenografts exceeded 1000 mm³ (T) or bleeding ulcers on the xenografts were observed (U).

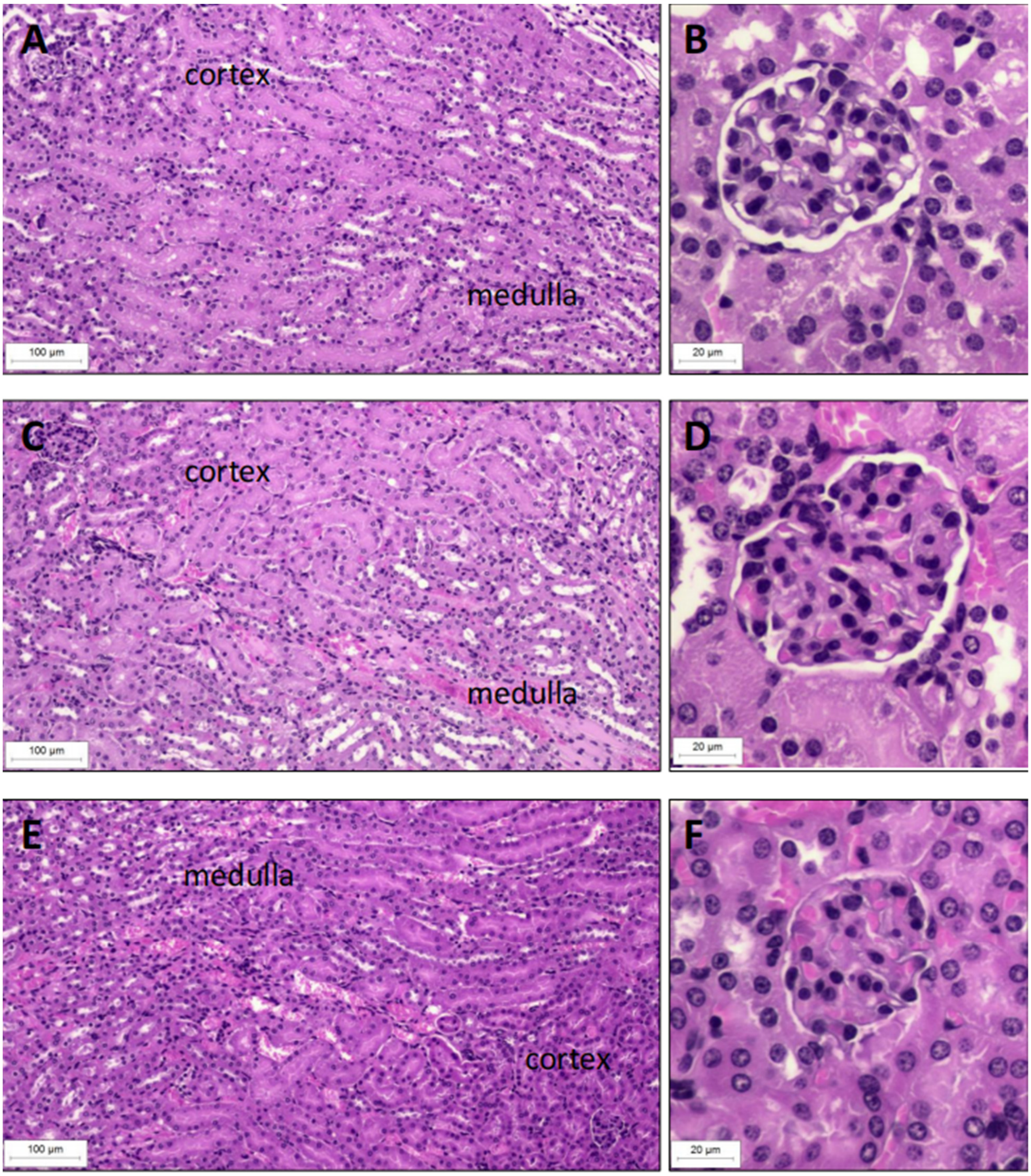

Figure S2. Results of pathological examination of kidneys. HE, 100x and 400x of kidney in mouse from (**A**,**B**) ADAPT6-ABD-mcDM1 group, (**C**,**D**) ADAPT$_{Neg}$-ABD-mcDM1 group and (**E**,**F**) PBS group.

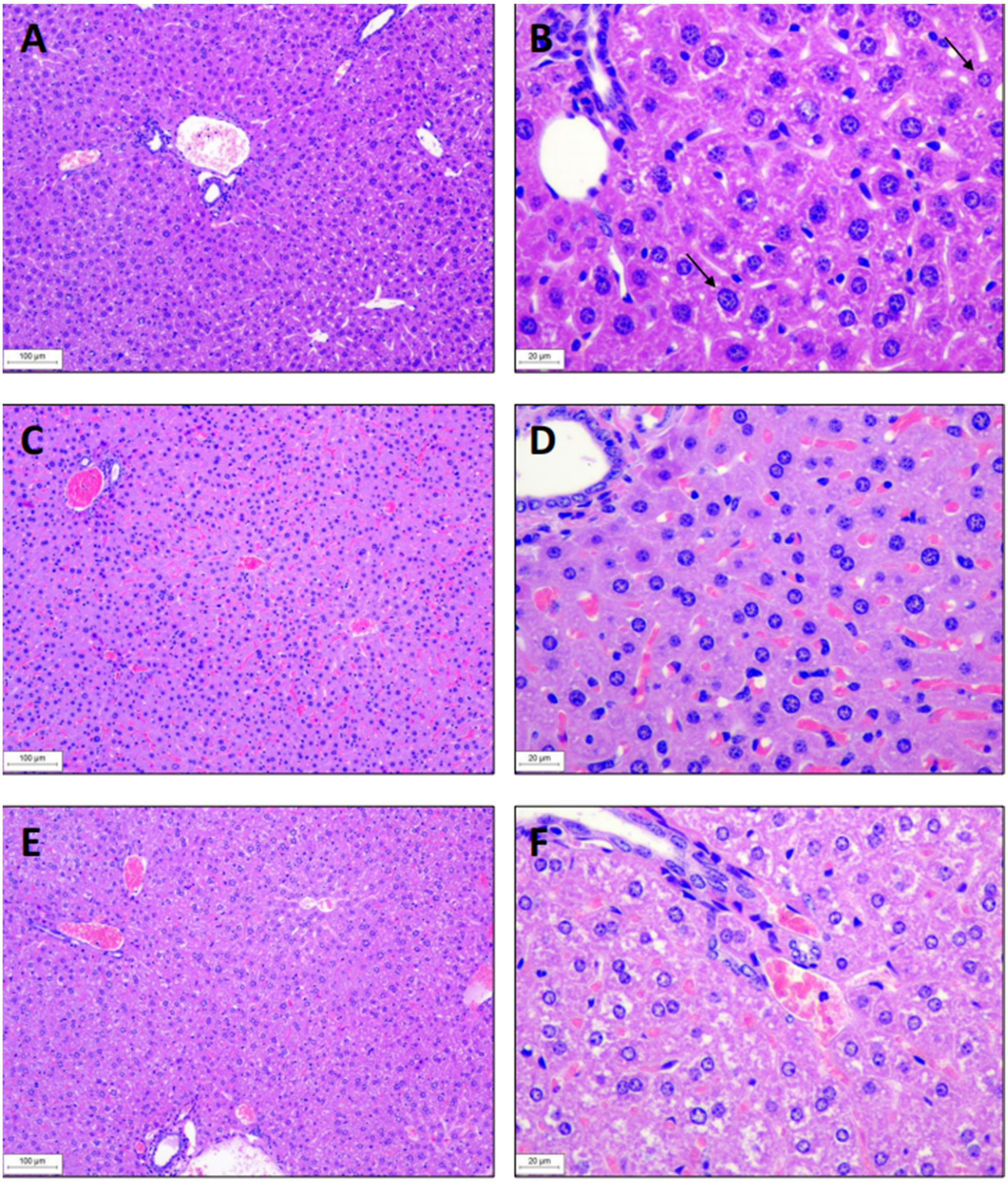

Figure S3. Results of pathological examination of livers. HE, 100x and 400x of liver in mouse from (**A**,**B**) ADAPT6-ABD-mcDM1 group (**C**,**D**) ADAPT$_{\mathrm{Neg}}$-ABD-mcDM1 group and (**E**,**F**) PBS group. Arrows indicate a small and a large nucleus.

Table S1. Tumor volume (mm^3) of ADAPT6-ABD-mcDM1 therapy experiments. Group A: ADAPT6-ABD-mcDM1; Group B: ADAPT$_{Neg}$-ABD-mcDM1; Group C: PBS

**Day**	**0**	**3**	**7**	**9**	**13**	**16**	**20**	**23**	**27**	**30**	**34**	**37**	**41**	**44**	**48**	**51**	**55**	**58**	**62**	**65**	**69**	**72**	**78**	**86**	**90**
**A1**	77	101	62	37	38	38	45	37	41	34	43	44	40	37	53	83	128	171	255	373	279	300	388	584	764
**A2**	98	141	75	66	67	78	86	93	71	98	96	110	126	153	216	207	250	304	314	324	287	310	399		
**A3**	95	132	86	63	54	56	55	34	24	31	32	36	31	35	51	55	70	103	121	117	118	156			
**A4**	63	84	57	52	46	55	65	61	86	71	68	85	112	133	157	246	195	225	225	243	177	220	233	328	374
**A5**	152	135	55	53	26	21	24	18	20	20	15	16	17	19	30	43	67	82	152	210	192	199	214	413	490
**A6**	96	124	91	71	94	82	69	65	72	79	103	107	109	154	182	279	268	242	258	234	168	226	325	437	512
**A7**	127	147	83	98	101	97	124	129	137	134	156	173	247	250	187	201	220	310	343	391	377	486			
**A8**	109	114	74	66	82	93	109	115	113	108	108	134	159	172	244	341	435	500	546	501	384	421			
**A9**	80	97	68	72	79	59	99	90	120	123	148	165	199	219	330	356	392	409	453						
**A10**	116	179	151	115	116	130	134	113	124	128	154	161	218	270	357	370	360	394	407	459	437	533			
**B1**	68	81	109	122	170	218	319	437	495																
**B2**	59	80	96	96	121	135	144	162	250	293															
**B3**	86	72	86	91	91	99	139	168	202	277	359	375	394												
**B4**	109	129	143	145	185	257	313	385	481	577															
**B5**	71	106	129	148	185	212	268	348	416	487	531	656													
**B6**	102	107	129	177	235	371	521	589																	
**B7**	95	121	177	233	296	361	366	403	527	502	502	502													
**B8**	145	155	238	271	327	411	521	566																	
**B9**	121	143	170	224	248	316	428	488	573																
**B10**	84	94	106	123	149	211	315	416																	
**C1**	83	94	146	135	146	159	167	185	230	262	279	287	306	233	215	231									
**C2**	98	68	79	86	96	107	124	137	257	272	386	529	637	759	978										
**C3**	56	53	53	73	85	95	134	156	330	407	672	767	809	791	838	982									
**C4**	63	102	169	189	221	316	526	550																	
**C5**	110	150	213	281	352	389	458	493	635																
**C6**	89	79	93	130	262	313	365	637	850	937	1000														
**C7**	82	108	155	128	182	173	258	324	367	362															
**C8**	69	59	75	107	117	128	197	222	312	407															
**C9**	91	117	156	243	346	455	572	778	911																
